# Bridging therapy is associated with improved cognitive function after large vessel occlusion stroke – an analysis of the German Stroke Registry

**DOI:** 10.1186/s42466-020-00079-9

**Published:** 2020-07-27

**Authors:** Philipp Ettelt, Ilko L. Maier, Marlena Schnieder, Mathias Bähr, Daniel Behme, Marios-Nikos Psychogios, Jan Liman, on behalf of the GSR-ET Collaborators

**Affiliations:** 1 Department of Neurology, Allgemeines Krankenhaus Celle, Celle, Germany; 2 Department of Neurology, University Medicine Göttingen, Göttingen, Germany; 3 Department of Neuroradiology, University Medicine Göttingen, Göttingen, Germany; 4 Department of Neuroradiology, Universitätsspital Basel, Basel, Switzerland

**Keywords:** Ischemic stroke, LVOS, Intravenous thrombolysis, Mechanical thrombectomy, Cognitive function

## Abstract

**Background:**

The targeted use of endovascular therapy (EVT), with or without intravenous thrombolysis (IVT) in acute large cerebral vessel occlusion stroke (LVOS) has been proven to be superior compared to IVT alone. Despite favorable functional outcome, many patients complain about cognitive decline after EVT. If IVT in addition to EVT has positive effects on cognitive function is unclear.

**Methods:**

We analyzed data from the German Stroke Registry (GSR, an open, multicenter and prospective observational study) and compared cognitive function 90 days after index ischemic stroke using MoCA in patients with independent (mRS ≤ 2 pts) and excellent (mRS = 0 pts) functional outcome receiving combined EVT and IVT (EVT + IVT) vs. EVT alone (EVT-IVT).

**Results:**

Of the 2636 GSR patients, we included 166 patients with mRS ≤ 2 at 90 days in our analysis. Of these, 103 patients (62%) received EVT + IVT, 63 patients (38%) were treated with EVT alone. There was no difference in reperfusion status between groups (mTICI ≥ 2b in both groups at 95%, *p* = 0.65). Median MoCA score in the EVT + IVT group was 20 pts. (18–25 IQR) vs. 18 pts. (16–21 IQR) in the EVT-IVT group (*p* = 0.014). There were more patients with cognitive impairment (defined as MoCA < 26 pts) in the EVT-IVT group (54 patients (86%)) compared to the EVT + IVT group (78 patients (76%)). EVT + IVT was associated with a higher MoCA score at 90 days (mRS ≤ 2: *p* = 0.033, B = 2.39; mRS = 0: *p* = 0.021, B = 4.38).

**Conclusions:**

In Patients with good functional outcome after LVOS, rates of cognitive impairment are lower with combined EVT and IVT compared to EVT alone.

**Trial registration:**

ClinicalTrials.gov Identifier: NCT03356392.

## Introduction

Ischemic stroke is one of the most frequent causes of permanent physical disability worldwide [1] and, in addition, is associated with an increasing incidence of cognitive impairment and dementia [2]. Even after good clinical recovery, about 30–50% of patients complain of cognitive impairment or dementia within the first year after an ischemic stroke [3–7]. In the course, the prevalence of dementia even increases [8] with a broad spectrum of cognitive disturbances, ranging from mild cognitive impairment into a manifest dementia [9–12]. Cognitive impairment not only reduces quality of life, but also increases mortality and health care costs [13]. The exact pathogenesis so far remains unclear. Since both ischemic stroke and cognitive impairment have an increasing prevalence in older age, the exact differentiation, especially of causality, is not easy. Pendlebury et al. could show a significant deterioration of cognitive function associated with an ischemic event in patients with already beginning (age-correlated-physiological) cognitive impairment [14]. Secondary analysis of the Framingham Heart Study conclude that post-stroke worsening in several dimensions of cognition cannot be explained solely by inferior cognitive performance before the stroke event or by concomitant common vascular risk factors [15]. One might assume that ischemic stroke causes a significant loss of cerebral substance with insufficient compensation due to the already aged “senile” brain. The functional “reserve” is lower in old age, which is also reflected in the risk factors for a post-stroke cognitive disorder: Here, in addition to the age (> 65 years) and already known cognitive impairment, cerebral atrophy in the temporal lobe, recurrent ischemic strokes, cardioembolic infarcts and so-called “white matter lesions” are mentioned [16–19].

Early recanalizing treatment of stroke reduces ischemic lesion burden. In addition to the treatment with thrombolysis using intravenous tissue plasminogen activator (rtPA, IVT), endovascular therapy (EVT) has been shown to be the gold standard in the acute phase and significantly improves functional outcome [20–24]. In addition, a recent study by López-Cancio et al. could demonstrate the benefit of EVT versus IVT alone for cognitive outcome parameters in a group of patients with good functional outcome (defined as a score on the modified Rankin Scale (mRS): ≤ 2) [25].

In the acute treatment of stroke IVT is often combined with EVT (“bridging-therapy”). Currently, the advantage of bridging therapy versus non-bridging is controversially discussed [26–29]. Recent studies indicate a possible benefit of bridging therapy in terms of recanalization rate and functional outcome, with no significant increase in the rate of intracranial hemorrhage in the bridging group [30, 31]. An analysis of data from the Virtual International Stroke Trials Archive (VISTA) study suggests that stroke patients may benefit from rtPA treatment in terms of cognition [32]. But it is still unclear whether bridging therapy also has positive effects on cognition compared to EVT alone.

In this study, we investigate the effects of bridging treatment (IVT + EVT) vs EVT alone (EVT-IVT) on cognitive function in patients with LVOS and good outcome.

## Material and methods

### Patient population and clinical characteristics

Available data of all patients enrolled in the German Stroke Registry – Endovascular Treatment (GSR-ET 07/2015–04/2018; ClinicalTrials.gov Identifier: NCT03356392) were included. The GSR-ET is an ongoing, open-label, prospective, multicenter registry of 25 sites in Germany, collecting consecutive patients undergoing EVT.

### Group classification

Initially, patients with an mRS between 0 and 2 were selected from the entire GSR database in this study. In a next step, only patients with a MoCA-evaluation after 90 days were included for further analysis. A Bridging- (EVT + IVT) and a Non-Bridging group (EVT-IVT) were compared. Subsequently, subgroups were formed for comparison, which were analyzed separately. This includes a group with a mRS of 0 points, as well as a subgroup formation by stroke-localization (right vs. left and anterior vs. posterior cerebral circulation).

To further differentiate the cognitive impairment (CI), patient groups were formed on the basis of the 90-day MoCA scores (0–9 pts.: severe CI; 10–17 pts.: moderate CI; 18–25 pts.: mild CI; > 25 pts. No CI). Although this classification is currently not based on scientific evidence, the homepage offers this classification as a possible graduation of the severity of a CI [33].

### Statistical analysis

The direct group comparison was done by descriptive statistics. Categorical sizes were given with mean and standard deviation, as well as absolute frequencies. Continuous variables were given by median, quartiles, minimum and maximum. Comparative test procedures between intervention and control groups were performed by chi-square test and non-parametric method (Mann-Whitney U test), depending on variable. Missing values in variables of the data set were determined with an in-depth data analysis. For continuous and categorical independent variables with more than 10% missing values, the multiple imputation feature implemented in SPSS was used to calculate the missing values using a regression model [34]. This method was used to counteract the bias of the result by complete-case analysis and to increase the validity of the study [35–37]. Potential confounders were filtered out by univariate pre-testing, with a *p*-value < 0.3 being considered predictive of the outcome “cognitive impairment”. For the continuous endpoint MoCA-value after 90 days, a linear regression model was performed. Finally, the two groups with possible confounders were further processed in a multivariate logistic regression model. All calculations were based on a 5% significance level.

Statistical analysis was performed using SPSS (version 26, IBM Corporation, Armonk, New York, USA), written with Word (Microsoft Corporation, Redmond, Washington, USA). Graphics were created using Excel (ibid).

## Results

### Baseline-characteristics

From the 2636 patients included in the GSR the 90-day MoCA had been recorded in 215 (8.1%) patients. Of these, 166 (77.2%) patients had an independent functional outcome with a 90-day mRS ≤ 2. In this group 103 (62%) patients were treated with EVT + IVT, 63 (38%) received an EVT alone. These patients most likely did not receive EVT due to various contraindications for IVT (24 (38%) patients had an unclear or exceeded time window with already beginning infarct signs in the imaging, 39 (62%) had an active anticoagulation, severe comorbidities as contraindication for IVT or recent surgery). The baseline characteristics of both groups are summarized in Table 1. In most cases stroke was located in the anterior cerebral circulation (*n* = 146, 88%); the remaining 20 (12%) patients had vertebrobasilar strokes. There was a significant shorter median symptom onset to admission time in patients with EVT + IVT compared to in the EVT-IVT group (69 min, IQR 40–171 min vs 205 IQR 73–508 min in the EVT-IVT group, *p* < 0.001). The proportion of smokers (37.1% vs. 27.3%, *p* = 0.19) and patients with anticoagulation (28,6% vs. 0%, *p* < 0.001) were higher in the EVT-IVT group compared to the EVT + IVT group. Functional impairment, quantified by the National Institute of Health Stroke Scale (NIHSS) on admission was more pronounced in the EVT + IVT group (12 pts., IQR 7–16 pts., vs. 10 pts., IQR 5–15 pts., *p* = 0.14) as well as the ASPECT score for the assessment of early infarct signs, which was also higher in the EVT + IVT group (9 pts., IQR 8–10 pts. vs. 8 pts., IQR 7–10, *p* = 0.01). There was no significant difference in groin to reperfusion time in both groups (37 min, IQR 22,5–56 min in the EVT + IVT-group and 34 min, IQR 26–55 in the EVT-IVT group; *p* = 0.922). At discharge, both groups showed mild symptoms with a median NIHSS of 2 points (*p* = 0.24).

**Table 1:** Baseline Characteristics MRS ≤ 2

Variable	Bridging	Non-Bridging	***p***-value
	(***n*** = 103)	(***n*** = 63)	
Sex (n, %)			0.29
Male	55 (53.4)	39 (61.9)	
Female	48 (46.6)	24 (38.1)	
Age (mean ± SD)	67 (±14.28)	69 (±11.18)	0.29
Vasc. risk-factors (n, %)			
Art. hypertension	69 (67)	45 (71.4)	0.55
Smoking	27 (27.3)	23 (37.1)	0.19
Diabetes	17 (16.5)	14 (22.2)	0.36
Dyslipidemia	39 (37.9)	23 (36.5)	0.86
Atrial fibrillation	25 (24.3)	21 (33.3)	0.21
Baseline medication (n, %)			
Aspirin	28 (28.9)	12 (20)	0.22
Clopidogrel	3 (3.1)	2 (3.3)	0.93
Anticoagulation	0 (0)	18 (28.6)	< 0.001
NIHSS at admission (median points, IQR)	12 (7–16)	10 (5–15)	0.14
Symptom-onset to admission (median time, IQR)	69 (40–171)	205 (73–508)	< 0.001
ASPECTS (median points, IQR)	9 (8–10)	8 (7–10)	0.01
General anesthesia (n, %)	68 (66)	41 (65)	0.16
Treatment adverse event (n, %)	12 (11)	8 (12)	0.72
NIHSS at discharge (median points, IQR)	2 (0–4)	2 (1–3)	0.24
mTICI ≥2b after EVT (n, %)	98 (95)	60 (95)	0.65
Stroke etiology (n, %)			
Large-artery atherosclerosis	22 (21)	16 (25)	0.57
Cardioembolism	37 (36)	27 (43)	0.41
Small-vessel occlusion	0 (0)	1 (1)	0.38
Stroke of other determined etiology	9 (9)	2 (3)	0.21
Stroke of undetermined etiology	23 (22)	15 (24)	0.85
Stroke localization (n, %)			
Right hemispheric stroke	56 (54)	29 (46)	0.44
Left hemispheric stroke	34 (33)	27 (43)	0.44
Vertebrobasilar stroke	13 (13)	7 (11)	0.99
A. cerebri anterior	1 (1)	0 (0)	0.99
A. cerebri media M1 proximal	33 (32)	28 (44)	0.14
A. cerebri media M1 distal	26 (25)	14 (22)	0.71
A. cerebri media M2	23 (22)	13 (21)	0.85
A. carotis interna T	10 (10)	5 (8)	0.79
A. carotis interna extracranial	2 (2)	2 (3)	0.64

*SD* standart deviation, *IQR* interquartile range

A subgroup of 54 (33.7%) patients had no stroke associated functional impairment corresponding to a 90-day mRS of 0 pts. The baseline characteristics of these patients are given in supplementary Table 1. This subgroup showed differences in gender distribution with a lower proportion of male patients in the EVT + IVT group compared to the EVT-IVT group (38.5% vs. 66.7%, *p* = 0.06). Again, there was a significant difference between the two groups in terms of median time between symptom onset and admission (82 min, IQR 45–236 min in the EVT + IVT group, 156 min, IQR 73–553 min in the EVT-IVT group, *p* < 0.001). Patients with bridging therapy also had a higher NIHSS on admission compared to EVT-IVT patients (NIHSS median 11 pts., IQR 7–15 in the EVT + IVT group vs. 6 pts., IQR 4–12 in the EVT-IVT group, *p* = 0.057), while the NIHSS was slightly higher at discharge in the EVT-IVT group (1 pt., IQR 0–2 in the EVT-IVT and 0 pts., IQR 0–2 in the EVT + IVT group, *p* = 0.101). Again, the proportion of anticoagulated patients in the EVT-IVT group was higher (33.3% vs. 0%, *p* < 0.001).

### MoCA-score at day 90

For patients with a 90-day mRS ≤ 2, there was a significant difference in the MoCA scores between the EVT + IVT and EVT-IVT group with a median MoCA score in the EVT + IVT group of 20 points (IQR 18–25 pts) vs 18 points (IQR 16–21 pts) in the EVT-IVT group (*p* = 0.014). For patients with excellent functional outcome (mRS = 0), we also found a significantly higher MoCA score in the EVT + IVT group (21 points (IQR 18–29 pts) vs 19 points (IQR 17–21 pts), *p* = 0.018).

After the adjustment for sex, age, NIHSS, symptom-onset to admission-time, ASPECTS, vascular-risk factors and baseline medication a significant association between EVT + IVT and higher MoCA-scores after 90 days persisted both for patients with independent- as well as excellent functional outcome (mRS ≤ 2: B = 2.39, 95%-CI = 0.20–4.58, *p* = 0.033; mRS = 0: B = 4.38, 95%-CI = 0.67–8.08, *p* = 0.021) (Table 2). This difference was independent concerning reperfusion status after endovascular treatment since 95% reached mTICI≥2b in both groups (*p* = 0.65).

**Table 2:** Influence of variables on the 90d MoCA value

Variable	Single linear regression		Multiple linear regression	
	B (95% CI)	***p***-value	B (95% CI)	***p***-value
**MRS ≤ 2**				
Bridging-therapy	2.01 (0.4–3.62)	0.015	2.39 (0.20–4.58)	0.033
Sex			0.52 (−1.08–2.12)	0.526
Age			−0.11 (−0.17−−0.04)	0.001
Symptom-onset to admission			0.00 (−0.003–0.003)	0.842
NIHSS at admission			-0.04 (−0.17–0.09)	0.551
Smoking			−1.91 (−3.71--0.11)	0.038
Atrial fibrillation			−0.60 (−2.71–1.51)	0.576
Aspirin premedication			−0.5 (−2.48–1.49)	0.623
Anticoagulation			1.91 (−1.23–5.05)	0.232
ASPECT-Score			−0.25 (− 0.94–0.43)	0.46
NIHSS at discharge			−0.09 (− 0.29–0.12)	0.405
**MRS = 0**				
Bridging-therapy	3.55 (0.78–6.31)	0.013	4.38 (0.67–8.08)	0.021
Sex			0.58 (−2.1–3.26)	0.669
Age			−0.07 (−0.21–0.07)	0.319
Symptom-onset to admission			−0.003 (− 0.76–1.07)	0.721
NIHSS at admission			−0.06 (− 0.24–0.13)	0.56
Smoking			0.80 (−2.50–4.11)	0.63
Dyslipidemia			2.49 (−0.28–5.27)	0.078
Aspirin premedication			−2.28 (−5.41–0.86)	0.154
Anticoagulation			−0.49 (−7.32–6.34)	0.881
NIHSS at discharge			0.16 (−0.76–1.07)	0.721

*CI* Confidence Interval

After correction for relevant confounders (supplementary Table 2), patients with strokes in the anterior circulation receiving combined EVT and IVT tended to show a higher MoCA-score compared to the EVT-IVT group (B = 2.29; *p* = 0.061; median MoCA 20 pts. (IQR 17–24 pts) vs 18 points (IQR 16–21 pts)). There were no significant differences in MoCA-scores between both groups in patients with strokes in the posterior circulation (median MoCA 21 pts., IQR 18–28 in the EVT + IVT vs 20 pts., IQR 14–27, in the EVT-IVT group; B = 2.12; *p* = 0.603) (supplementary Table 3).

### Cognitive impairment

Overall, 133 of the 166 patients included in the analysis (80%) showed cognitive impairment (defined as a MoCA-score < 26 points) 90 days after the index event. The rate was 76% in the EVT + IVT and 86% in the EVT-IVT group. However, this difference was not statistically significant after correction for possible confounders (*p* = 0.225; OR: 2.27). In the group with excellent functional outcome (mRS = 0) the prevalence of cognitive impairment was significantly lower (66%) in the EVT + IVT group, compared to the EVT-IVT group (93%; *p* = 0.034; OR: 23.15; suppl. Table 1). In the subgroup analysis on individual examination of the stroke localization, there was no significant difference between the prevalence of cognitive impairment between the EVT + IVT and EVT-IVT group (suppl. Table 4).

### Severity of cognitive-impairment

Rates of moderate and severe cognitive impairment were significantly higher in the EVT-IVT (25 of 63 patients; 39.6%) versus the EVT + IVT group (25 of 103 patients, 24%; *p* = 0.040, OR 3.38). In patients with mRS = 0, there also was a significant higher risk for moderate to severe cognitive impairment at 90 days with a prevalence of 13% in the EVT + IVT- and 33% in the EVT-IVT group (*p* = 0.035, OR: 0.08) (Table 3).

**Table 3:** Severity of Cognitive Impairment

	Bridging-group	Non-Bridging group	OR (95% CI)	***p***-value
	(n, %)	(n, %)		
**mRS ≤ 2**				
No Cognitive Impairment	25 (24.3)	9 (14.3)	0.52 (0.23–1.20)	0.126
Mild Cognitive Impairment	53 (51.4)	29 (46)	0.66 (0.27–1.60)	0.355
Moderate Cognitive Impairment	25 (24.3)	24 (38.1)	0.38 (0.15–0.97)	0.042
Severe Cognitive Impairment	0 (0)	1 (1.5)	–	–
**mRS = 0**				
No Cognitive Impairment	13 (33.3)	1 (6.6)	0.14 (0.02–1.21)	0.074
Mild Cognitive Impairment	21 (53.8)	9 (60)	0.18 (0.02–1.59)	0.122
Moderate Cognitive Impairment	5 (12.8)	5 (33.3)	0.08 (0.01–0.83)	0.035
Severe Cognitive Impairment	0 (0)	0 (0)	–	–

Cognitive Impairment (CI): No CI: MoCA ≥ 26 pts., Mild CI: MoCA > 17 pts., Moderate CI: MoCA > 10 pts., Severe CI: MoCA < 10 pts.

## Discussion

In the present study, we found lower rates of cognitive impairment in LVOS-patients treated with EVT + IVT compared to patients with EVT alone. EVT + IVT patients performed better in the 90-day MoCA compared to patients treated with EVT alone, even though the initial neurological deficits were more pronounced in the EVT + IVT group. Concerning the similar reperfusion rates in both groups and the correction for symptom onset to admission time in our analysis, IVT seems to have a positive effect not only on functional outcome-, but also on cognition after stroke. In particular, IVT seems to have the highest effect in patients with excellent functional outcome. The effect of rtPA on cognition has so far been poorly understood, and the few post-stroke studies with cognitive endpoints showed heterogeneous results [38]. This is probably due to the different test methods used. At least until now, a benefit of the rtPA therapy with regard to visuoconstrictive abilities could be recognized [39].

Comparison of baseline characteristics between the EVT + IVT and the EVT-IVT group showed a significant difference in onset-to-admission times. In the EVT + IVT group 25% of patients have been treated within 40 min after symptom-onset, which, considering the findings of this study, underlines the importance to minimize onset-to-admission times to achieve not only favorable functional-, but also favorable cognitive outcomes.

In our study, we found a high rate of CI after 90 days in general (80%), which is well above the expected prevalence in other studies [3, 4, 7]. A selection-bias could occur due to the limited group size in this study and therefore could limit the generalizability of our data, as only around 8% of patients included in the GSR received cognitive testing. This low rate is most likely to be explained by the mere necessity of a personal follow-up visit, which certainly results in a high proportion of drop-outs. Furthermore, patients with post stroke aphasia or pre-existing CI could not be entirely excluded from the study population. Both pre-stroke cognitive impairment and post stroke aphasia are major confounders concerning post-stroke cognitive function and lack of correction for these factors represents a major limitation of our study. However, one could argue that it is likely that only patients underwent MoCA-testing being judged eligible to perform the tasks by the rater and that patients with significant aphasia and pre-stroke cognitive impairment should not have been rated as having a 90 days mRS ≤ 2. In addition, the time span of 90 days between cognitive testing and index event is very tight for the assessment of a stroke-related CI. Other studies suggest an interval of at least 6 months to even speak of post-stroke dementia [12]. However, an increased incidence of CI in the first period following a stroke or even TIA has been described in other studies [6, 8, 40–42]. Another bias in this study might occur due to the lack of cognitive testing at baseline, so no definitive statement on the incidence of CI, but only on prevalence in the course after stroke event can be made. Nonetheless, the prevalence remains considerable, bearing in mind that it refers to a patient population with good functional outcome.

A particular advantage in terms of cognition at different stroke localization could not be demonstrated. At least we could show that EVT + IVT did not yield any difference in terms of cognitive outcome in patients with stroke located in the posterior cerebral circulation. This also seems understandable when considering the brain region which is provided for by the vertebrobasilar vessels. Especially the neuropsychological and higher cognitive performance, which are queried by the testing via MoCA, are supplied with blood by the anterior cerebral circulation.

The conspicuous link between poorer MoCA testing in smokers in this study has already been observed in larger studies [43, 44] and underlines the importance of nicotine abstinence as a modifiable risk factor in the post-stroke situation. Furthermore, the obvious correlation between higher age and worse performance in the MoCA test is understandable, as the prevalence of CI increases in older age [45, 46].

The classification of CI into different categories (none, mild, moderate and severe CI) based on the MoCA score has yet not been validated in any study. Thus, this classification should be interpreted with an appropriate restraint. Further studies to evaluate a possible classification of CI after stroke with corresponding cut-off values in MoCA are desirable. In spite of these facts, considering the distribution of points in the individual groups, it is noticeable that significantly inferior cognitive performance (defined as a MoCA value below 17 points) occurs less frequently in the EVT + IVT than in the EVT-IVT group. This should be taken into account in the discussion about the potential benefits of a bridging therapy in the acute treatment of LVOS.

Our study is limited by various factors. On the one hand, the lack of cognitive baseline testing means that no real statement can be made about the incidence of cognitive disorders after mechanical thrombectomy. In addition, patients with pre-existing cognitive impairment or even dementia could distort the overall result. On the other hand, a further measurement using the MoCA-test in the long term would have been desirable to assess the development of cognitive impairments. In future studies, in addition to evaluating cognitive status before enrollment (e.g. by means of a simple questionnaire like the Informant Questionaire for Cognitive Decline in the Elderly IQCODE [47]), a simple follow-up should be considered to minimize the drop-out rate. An improvement in data collection could possibly be achieved by using the telephone version of the MoCA (t-MoCA), which has also been validated in clinical studies and described as very sensitive [48, 49]. In addition to the major confounders being accounted for in the analysis of this study, there are many other factors influencing cognitive function after stroke like history of previous stroke, history of pre-stroke cognitive decline and postprocedural complications. In this study, it was not possible to take these factors into account, representing a major limitation. Another limitation of this study, besides the low number of cases and possible drop-out rate with the risk of selection-bias, is the retrospective, non-randomized study design. In contrast, data was collected prospectively and with a face-to-face interview in the included patients and were collected in multiple large thrombectomy-centers throughout Germany, representing reliable real word data.

## Conclusion

Our data point to a possible benefit of bridging therapy in the controversy between bridging and non-bridging approaches. Even if the median MoCA value differs only by 2 points between the two groups after 90 days, at least this indicates a trend, which should be further verified in larger-scaled, prospective designed studies. In addition, our findings highlight the importance of routine cognitive testing in patients with favourable outcome after LVOS.

## Supplementary information

**Additional file 1 Suppl. Table 1**. Influence of variables on Cognitive Impairment after 90 days.

**Additional file 2 Suppl. Table 2**. Relevant subgroup differencies.

**Additional file 3 Suppl. Table 3**. Influence of variables in the subgroups on the 90d MoCA value.

**Additional file 4 Suppl. Table 4**. Influence of variables on Cognitive Impairment (MoCA < 26 pts) after 90 days.

## Data Availability

The data that support the findings of this study are available from the GSR-ET Collaborators but restrictions apply to the availability of these data, which were used under license for the current study, and so are not publicly available. Data are however available from the authors upon reasonable request and with permission of the GSR-ET steering committee.

## Abbreviations

ASPECTS: Alberta stroke programme early CT score
CI: Cognitive Impairment
EVT: Endovascular therapy
GSR: German Stroke Registry
IQR: Interquartile range
IVT: Intravenous thrombolysis
LVOS: Large cerebral vessel occlusion stroke
MoCA: Montreal Cognitive Assessment
mRS: Modified Rankin Scale
mTICI: Modified treatment in cerebral ischemia score
NIHSS: National Institutes of Health Stroke Scale
OR: Odds Ratio
rtPA: Recombinant tissue plasminogen activator
VISTA: Virtual International Stroke Trials Archive
